# The development of a classification system for the use of the (modified) side-shift approach to conservative management of scoliosis

**DOI:** 10.1186/1748-7161-8-S1-P19

**Published:** 2013-06-03

**Authors:** T Betts

**Affiliations:** 1 Royal National Orthopaedic Hospital , London, UK

## Background

Classification systems exist to guide the management of surgery (the King and Lenke systems) and Brace treatments (Chêneau, Rigo and SpineCor). Few exist for the management of physical exercise (Lehnerth-Schroth classification system). The side shift approach to correction of scoliosis curves has been used by therapist at the RNOHT for over15 years. The side shift approach was developed by Mrs Min Mehta, and has been Modified using consensus based evidence of SOSORT. Clinical observations had indicated that not all patients could actively (Auto) correct to beyond the trunk midline, a key principle of Side Shift. At the RNOHT a classification system based upon the ability of an individual to auto-correct the spine during a side shift movement has been developed to aid the appropriate application of the shift exercises, and allow future comparative analysis. Consecutive patients who have AIS, seen by the author and 1 colleague in the calendar year of 2011, were tested for the application of the Side-Shift Classification System. Three types of Side-Shift were developed.

## Aim

To develop a Clinical Classification System for Physical Therapy. To demonstrate if clinical spinal mobility correlates with indications of Side-Shift exercises.

## Methods

Types of Side-Shift Correction are being analysed and compared to diagnosed Curve types, measurements are being recorded of Cobb Angle and Hypermobility Scores (using Beighton Hyperlaxity scale) and scoliometer measurements. Database statistics are also being recorded. Inter-observer and Intra-observer reliability is to be measured to demonstrate the consistency of the Classification System.

## Results

To date too few consecutive patients have been collated to be able to provide numerable results. The Author aims to present Validation results after comparing thirty consecutive AIS patients. Currently, 13 consecutive patients have been reviewed by two Physical Therapist, who have independently categorized the Side Shift Type of each presenting patient.

## Conclusions

The author presents this article as a preliminary study in to the development of the Side Shift Classification system. The author requests analysis into the usefulness of the scale, and advice on future research and development. Interim analysis suggests that this is a useful descriptive basis for classifying Side Shift mobility in a population with Scoliosis.

## References

[B1] Lenke LG Betz RR Harms J Bridwell KH Clements DH Lowe TG Blanke K Adolescent idiopathic scoliosis: a new classification to determine extent of spinal arthrodesis J Bone Joint Surg Am 2001 83-A 8 1169 1181 11507125

[B2] M MH Active Correction by Side-Shift : An alternative treatment for early idiopathic scoliosis 1985 New York: Praeger

[B3] Rigo MD Villagrasa M Gallo D A specific scoliosis classification correlating with brace treatment: description and reliability Scoliosis 5 1 1 20205842 10.1186/1748-7161-5-1 PMC2825498

